# Impaired Renal Mitochondria and Bioenergetics During Obesity-Associated NAFLD

**DOI:** 10.3390/nu18132061

**Published:** 2026-06-24

**Authors:** Amod Sharma, Reza Hakkak, Shannon Rose, Neriman Gokden, Nirmala Parajuli

**Affiliations:** 1 Department of Pharmacology and Toxicology, University of Arkansas for Medical Sciences, Little Rock, AR 72205, USA; asharma2@uams.edu; 2 Department of Pediatrics, University of Arkansas for Medical Sciences, Little Rock, AR 72205, USA; rhakkak@uams.edu; 3 Department of Dietetics and Nutrition, University of Arkansas for Medical Sciences, Little Rock, AR 72205, USA; 4 Arkansas Children’s Research Institute, Little Rock, AR 72202, USA; 5 Department of Health Promotion and Disease Prevention, College of Nursing, The University of Tennessee Health Science Center, Memphis, TN 38103, USA; srose37@uthsc.edu; 6 Department of Pathology, University of Arkansas for Medical Sciences, Little Rock, AR 72205, USA; gokdenneriman@uams.edu

**Keywords:** obesity–NAFLD, kidney injury, mitochondrial dynamics, ATP, metformin

## Abstract

**Background/Objectives:** Obesity-associated non-alcoholic fatty liver disease (NAFLD) drives systemic metabolic stress and accelerates chronic kidney disease, yet the mechanistic links remain unclear. Mitochondrial dysfunction has emerged as a central mediator of obesity-induced organ injury. Here, we investigated renal mitochondrial remodeling in a rat model of obesity-associated NAFLD (Ob-NAFLD) and examined the effects of metformin. **Methods:** Female Zucker rats (obese fa/fa and lean Fa/Fa) were fed an AIN-93G diet for eight weeks, followed by 10 weeks of metformin treatment in designated groups. Kidney tissues were analyzed using biochemical assays, immunoblotting, blue native PAGE, in-gel activity assays, and histological evaluation. **Results:** In Ob-NAFLD rats, renal ATP levels were elevated despite reduced electron transport chain (ETC) Complex III and increased Complex V expression, reflecting compensatory ATP synthase hyperactivity uncoupled from efficient oxidative phosphorylation. Mitochondrial dynamics were disrupted such that inhibitory phosphorylation of DRP1 was reduced, promoting fission, and total OPA1 expression was decreased with a shift in short-to-long isoform balance, indicating impaired fusion and cristae remodeling. Notably, ATPase inhibitory factor 1 (IF1), a checkpoint that limits ATP synthase overdrive, remained stably expressed, suggesting an adaptive ceiling or failed protective control under chronic metabolic stress. Metformin partially alleviated bioenergetic stress by lowering ATP and modestly restoring Complex III, yet ETC imbalance and structural remodeling persisted, revealing the limitations of metabolic modulation alone. **Conclusions**: These findings position entrenched mitochondrial dysregulation as a mechanistic bridge linking obesity-driven liver disease to kidney injury. Therapeutic strategies combining metabolic interventions with targeted restoration of ETC coordination, mitochondrial dynamics, and regulatory checkpoints such as IF1 may be required to fully restore renal mitochondrial health and prevent the progression of metabolic kidney disease.

## 1. Introduction

Obesity represents a major global health burden and is the primary driver of non-alcoholic fatty liver disease (NAFLD), which currently affects approximately 25% of the global adult population [1]. While traditionally viewed as a liver-specific disorder, NAFLD is now recognized as a multi-systemic disease with significant extrahepatic complications, most notably chronic kidney disease (CKD) [2,3]. Clinical and experimental studies suggest that obesity and NAFLD independently and synergistically contribute to renal injury and functional decline [4,5].

The kidney, particularly the renal proximal tubule, is one of the most metabolically active tissues in the body. Proximal tubular epithelial cells depend predominantly on mitochondrial oxidative phosphorylation (OXPHOS) to meet the high ATP demands required for solute reabsorption and cellular homeostasis [6,7]. In contrast to many other renal cell types, proximal tubular cells have limited glycolytic capacity, making them highly dependent on fatty acid oxidation as a primary energy source [8,9,10]. This imbalance leads to increased electron transport chain (ETC) flux, mitochondrial reactive oxygen species (ROS) generation, and impaired coupling efficiency. Over time, these alterations disrupt the balance between OXPHOS and glycolysis, contributing to bioenergetic stress, mitochondrial damage, and premature cellular senescence [8,11,12,13,14]. This metabolic specialization renders them particularly vulnerable to conditions of lipid overload and mitochondrial dysfunction.

Central to OXPHOS is the mitochondrial electron transport chain (ETC), composed of five multi-subunit complexes (Complexes I–V) embedded in the inner mitochondrial membrane. Electrons derived from nutrient oxidation are transferred through Complexes I–IV to molecular oxygen, generating water, while proton pumping establishes an electrochemical gradient that drives ATP synthesis via Complex V (ATP synthase) [15,16]. Although efficient, ETC activity is intrinsically associated with electron leakage, primarily at Complexes I and III, leading to the formation of reactive oxygen species (ROS) [17,18,19]. Thus, ETC activity not only determines ATP availability but also directly influences cellular redox balance and mitochondrial integrity.

In obesity, chronic nutrient excess and lipid accumulation impose severe metabolic stress on mitochondria, leading to electron transport chain (ETC) dysfunction, dysregulated ATP production, and excessive generation of ROS [20,21]. Consequently, impaired mitochondrial bioenergetics has emerged as a critical mechanistic link between metabolic overload and kidney injury, driving tubular damage, inflammation, and progressive loss of renal function [22,23,24].

Mitochondrial health is governed by mitochondrial dynamics, which is defined as the coordinated cycles of fission and fusion that preserve mitochondrial integrity and metabolic adaptability [25,26]. Dynamin-related protein 1 (DRP1) is a central mediator of mitochondrial fission; its activation, often through the reversal of inhibitory phosphorylation, promotes excessive fragmentation and oxidative stress [14,27]. Conversely, optic atrophy protein 1 (OPA1) regulates mitochondrial fusion and cristae architecture. Under stress, OPA1 undergoes proteolytic processing that disrupts inner membrane structure and impairs ETC efficiency [28,29]. In obesity-related metabolic disorders, a shift toward excessive fission and disrupted fusion has been closely correlated with organ injury [24,30]. This sustained impairment further facilitates the activation of intrinsic apoptotic pathways. Structural disruption of mitochondrial membranes promotes the release of pro-apoptotic factors, such as cytochrome c, which triggers executioner of caspases, including caspase-3 [31]. Indeed, recently published work has demonstrated increased expression of cleaved caspase-3 during obesity-induced renal injury, underscoring the role of mitochondrial-driven pathways in organ damage [5,24].

Metformin, a first-line antidiabetic agent, exerts pleiotropic metabolic benefits beyond glycemic control, including improvements in hepatic steatosis, inflammation, and fibrosis in NAFLD [32,33]. At the cellular level, by activating AMP-activated protein kinase (AMPK), inhibiting complex I of the ETC, and attenuating oxidative stress [33,34]. While metformin is known to influence mitochondrial respiratory function and dynamics in various tissues [35,36,37], its specific impact on renal mitochondrial health within the obesity–NAFLD–chronic kidney disease axis remains incompletely defined. Specifically, it is unclear whether metformin preserves ETC integrity and regulates the DRP1/OPA1 balance in the context of obesity-induced renal pathology.

Importantly, our previous work demonstrated that obesity-associated NAFLD is accompanied by renal oxidative stress, tubular injury, and activation of mitochondrial-dependent apoptotic pathways, as evidenced by increased cleaved caspase-3 expression [5]. While metformin partially attenuated inflammatory and oxidative stress markers, it did not fully reverse renal structural damage, suggesting the involvement of persistent mitochondrial dysfunction in disease progression. However, these observations do not establish a direct causal role for NAFLD in kidney injury, as obesity itself represents an upstream systemic metabolic disturbance.

Based on this background, the present study used a rat model of obesity-associated NAFLD [32,38] to characterize renal mitochondrial alterations within a systemic metabolic disease context. Specifically, we examined changes in ETC complex integrity, mitochondrial bioenergetics, and key regulators of mitochondrial dynamics (DRP1 and OPA1). We further evaluated the effects of metformin on renal ATP production and mitochondrial protein expression in obese and lean animals. This study provides mechanistic insight into renal mitochondrial dysfunction in obesity-associated metabolic disease and highlights mitochondrial pathways as potential therapeutic targets in metabolic kidney injury.

## 2. Materials and Methods

### 2.1. Experimental Design

The experimental design and kidney tissue samples used in this study originated from previously published work [5,32,39]. Briefly, five-week-old female Zucker rats, including obese (fa/fa; *n* = 12) and lean (Fa/Fa; *n* = 12) genotypes, were purchased from Envigo and housed individually with ad libitum access to food and water. Female rats were selected to maintain experimental consistency and reduce variability related to sex-dependent differences in body weight gain, hormonal fluctuations, and metabolic responses that could confound early mechanistic assessment of mitochondrial dysfunction. All animals were fed an AIN-93G diet (Envigo) for eight weeks to induce obesity and non-alcoholic fatty liver disease (Ob-NAFLD), as previously described. Next, rats were randomly assigned to one of four experimental groups (*n* = 6 per group): Lean Control (Lean), Lean + Metformin (Lean + M), Obese-NAFLD (Ob-NAFLD), and Ob-NAFLD + Metformin (Ob-NAFLD + M). Metformin was administered for ten weeks by incorporating 1 g of metformin per kilogram of diet. At the end of the treatment period, animals were euthanized using 30% CO_2_ inhalation prior to decapitation. Kidney tissues were then harvested and processed for subsequent histopathological and biochemical analyses.

All animal procedures were approved by the Institutional Animal Care and Use Committee (IACUC) of the University of Arkansas for Medical Sciences and the Arkansas Children’s Research Institute (Protocol No. 3968; approved on 20 December 2019). The study was conducted in accordance with the guidelines of the United States Department of Agriculture (USDA, Washington, DC, USA) Animal Welfare Act.

### 2.2. Western Blot Analysis

Western blot analysis was performed using rat kidney homogenates [40]. Briefly, protein extracts were prepared in RIPA buffer, and equal amounts of protein (25 µg per sample) were separated on 4–12% gradient SDS–PAGE gels (Thermo Fisher Scientific, Waltham, MA, USA). Proteins were subsequently transferred onto polyvinylidene difluoride (PVDF) membranes for immunodetection. Membranes were incubated with the appropriate primary antibodies, followed by horseradish peroxidase-conjugated secondary antibodies (Table 1). Immunoreactive bands were visualized using an enhanced chemiluminescence detection kit (Thermo Fisher Scientific, Waltham, MA, USA; 34580) and captured with the iBright™ CL1500 Imaging System (Thermo Fisher Scientific, Waltham, MA, USA). Band intensities were quantified by densitometric analysis using AlphaEase FC software (3.1.2), and protein expression levels were normalized to VDAC (for mitochondrial proteins) and GAPDH.

### 2.3. Immunohistochemistry Analysis

Paraffin tissue blocks sliced at 4 µm thickness were mounted on a glass slide and deparaffinized with xylene and a series of graded ethanol washes [41]. Other kidney cross-sections underwent antigen retrieval via heating in sodium citrate buffer (pH 6.0) and sections were quenched with BLOXALL™ Endogenous Peroxidase and Alkaline Phosphatase Blocking Solution (Vector Laboratories, Newark, NJ, USA; SP-6000-100). The slides were blocked with blocking solution (3% BSA and 0.5% non-fat dry milk in TBS) for 20 min at room temperature and incubated overnight at 4 °C with primary antibodies. The antibodies and dilution used are listed in Table 1. Immunoreactivity was detected with ImmPRESS^®^ Polymer Detection Kit and reagent (Vector Laboratories, Newark, NJ, USA; MP-7451). All images were taken on a Nikon Eclipse Ni microscope with Nikon Elements software (BR 6.02.01).

### 2.4. ATP Assay

Renal ATP levels were quantified using a luminescence-based ATP Determination Kit (Thermo Fisher Scientific, Waltham, MA, USA; A22066) according to the manufacturer’s instructions. Kidney tissues (20 mg) were homogenized on ice using a luciferase-compatible lysis buffer. A freshly prepared 1X lysis buffer was generated by diluting a 20X stock solution composed of 200 mM Tris (pH 7.5), 2 M NaCl, 20 mM EDTA, and 0.2% Triton X-100. Tissue homogenates were incubated on ice for 10 min and subsequently centrifuged at 12,000× *g* for 10 min at 4 °C. The resulting supernatants were collected for ATP measurement. A total of 15 µg protein was used for each sample for the assay.

ATP content was determined based on the luciferin–luciferase reaction, in which ATP-dependent oxidation of luciferin generates luminescence proportional to ATP concentration. Samples and ATP standards were incubated with the luciferase reaction mixture, and luminescence was measured using a microplate luminometer. An ATP standard curve was generated for each assay, and ATP concentrations were calculated accordingly. All samples were assayed in triplicate, and experiments were performed under identical conditions to minimize inter-assay variability.

### 2.5. Blue Native Polyacrylamide Gel Electrophoresis (BN-PAGE) for Analysis of Mitochondrial Protein Complexes

Mitochondria were isolated from renal tissue by differential centrifugation, and protein concentration was determined using the BCA assay. Mitochondrial proteins were solubilized in Mitochondria Extraction Buffer (MEB), followed by the addition of 10% lauryl maltoside solution (maltoside-to-protein ratio = 2.5 g/g), incubated on ice for 20 min, and centrifuged at 20,000× *g* for 20 min at 4 °C [42]. A total of 11 µg protein was loaded onto pre-cast 3–12% Bis-Tris native gels (Thermo Fisher Scientific, Waltham, MA, USA; BN2011BX10). Electrophoresis was performed at 4 °C using dark blue cathode buffer initially, followed by light blue cathode buffer until completion. Proteins were either transferred onto PVDF membranes for immunoblotting with specific primary antibodies or subjected to in-gel activity staining. Band intensities were quantified using AlphaEase FC software (3.1.2) and normalized to control values.

### 2.6. In-Gel ATP Synthase (Complex V) Activity Assay

In-gel ATP synthase activity was assessed following Blue Native-PAGE separation of mitochondrial protein complexes. After electrophoresis, gels were briefly rinsed with distilled water and incubated in Tris-glycine buffer (35 mM Tris-HCl (pH 7.4), 270 mM glycine) for 20 min at room temperature. The activity reaction was initiated by incubating gels in Tris-glycine buffer containing 14 mM magnesium sulphate, and 0.2% lead nitrate, supplemented with ATP (5 mM final concentration). Gels were then incubated at 37 °C for 3 h until visible white precipitated bands corresponding to ATP hydrolysis activity appeared. Band visualization was enhanced by the addition of 1% ammonium thiosulfate, which improved contrast and stabilized the precipitated reaction product. The reaction was stopped by washing gels with distilled water. Gels were imaged, and band intensities were quantified using AlphaEase FC (3.1.2) and normalized to control samples.

### 2.7. Statistical Analysis

Data are expressed as mean ± standard error of the mean (SEM). Normality of data distribution was assessed using the Shapiro–Wilk test. Comparisons among multiple groups were performed using two-way analysis of variance (ANOVA). When normality assumptions were satisfied, Tukey’s post hoc test (α = 0.05) was applied for pairwise comparisons. For data that did not follow a normal distribution, the Mann–Whitney U test was used to compare differences between two groups. Statistical analyses and graphical representations were generated using GraphPad Prism (version 10.0.2). A *p*-value < 0.05 was considered statistically significant.

## 3. Results

In our previous work, Ob-NAFLD rats exhibited pronounced renal oxidative stress and prominent tubular injury, accompanied by elevated cleaved caspase-3, consistent with the activation of mitochondrial-mediated apoptotic pathways [5]. These findings suggested that mitochondrial dysfunction might have contributed to Ob-NAFLD-associated renal injury. To further define the structural and bioenergetic basis of this dysfunction, and to determine whether metformin modifies these alterations, we examined the expression of ETC complexes, ATP levels, and regulators of mitochondrial dynamics and ATP production in the kidney.

### 3.1. Obesity-Associated NAFLD Induces Selective Impairment of Renal ETC with Partial Modulation by Metformin

Renal tubular cells rely heavily on mitochondrial oxidative phosphorylation to meet their high ATP demands. This process depends on the coordinated function and structural integrity of the five ETC complexes located in the inner mitochondrial membrane [15,16]. To comprehensively evaluate renal mitochondrial ETC alterations in Ob-NAFLD-associated CKD, we assessed representative subunits from each ETC complex using SDS-PAGE Western blotting and immunohistochemistry.

**Figure 1:**
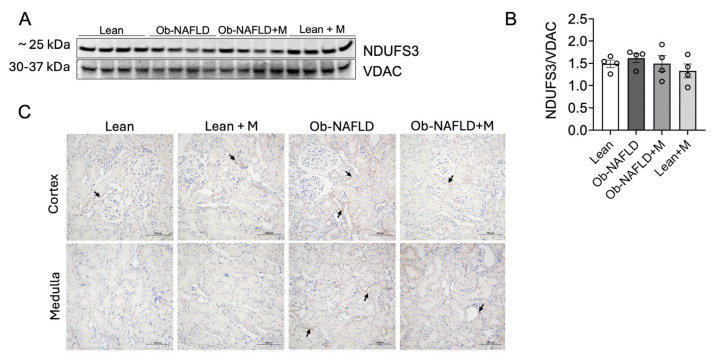
Renal mitochondrial Complex I protein (NDUFS3 subunit) expression in the kidneys of Ob-NAFLD rats with or without metformin treatment. (**A**) Representative Western blot images showing renal expression of NDUFS3 in Lean, Ob-NAFLD, Ob-NAFLD+M, and Lean+M groups (*n* = 4 per group). VDAC was used as the mitochondrial loading control. (**B**) Densitometric quantification of NDUFS3 protein expression normalized to VDAC. Data are presented as mean ± SEM. Each dot represents an individual animal. (**C**) Representative immunohistochemical staining of renal cortex and medulla sections showing NDUFS3 expression across study groups. Brown staining indicates positive immunoreactivity (arrows). Scale bars = 100 µm.

**Figure 2:**
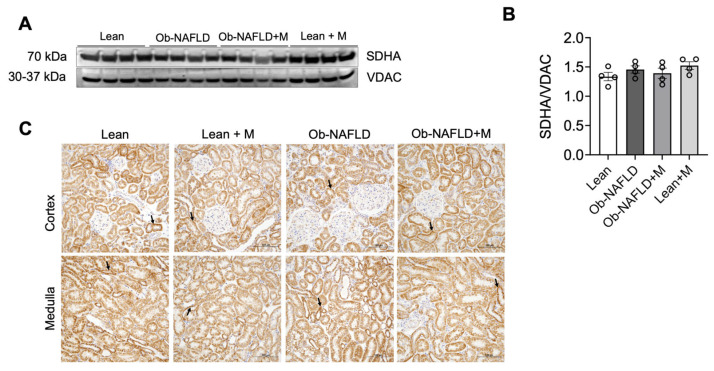
Renal mitochondrial Complex II (SDHA subunit) protein expression in the kidneys of Ob-NAFLD rats with or without metformin treatment. (**A**) Representative Western blot images showing renal expression of SDHA (Complex II subunit) in Lean, Ob-NAFLD, Ob-NAFLD+M, and Lean+M groups (*n* = 4 per group). VDAC was used as the mitochondrial loading control. (**B**) Densitometric quantification of SDHA protein expression normalized to VDAC. Data are presented as mean ± SEM. Each dot represents an individual animal. (**C**) Representative immunohistochemical staining of renal cortex and medulla sections showing SDHA expression across experimental groups. Brown staining indicates positive immunoreactivity (arrows). Scale bars = 100 µm.

Complexes I and II, which mediate the electron entry into the ETC and link tricarboxylic acid cycle activity to oxidative phosphorylation, were largely preserved (Figure 1 and Figure 2). Protein levels of Complex I (NDUFS3) and Complex II (SDHA) were comparable among lean controls, obese rats with NAFLD, and metformin-treated lean and obese groups (Figure 1A,B and Figure 2A,B). Immunohistochemical analysis of kidney sections further demonstrated consistent tubular localization of NDUFS3 and SDHA proteins across all groups (Figure 1C and Figure 2C), indicating preservation of upstream electron transfer despite Ob-NAFLD-associated kidney injury.

**Figure 3:**
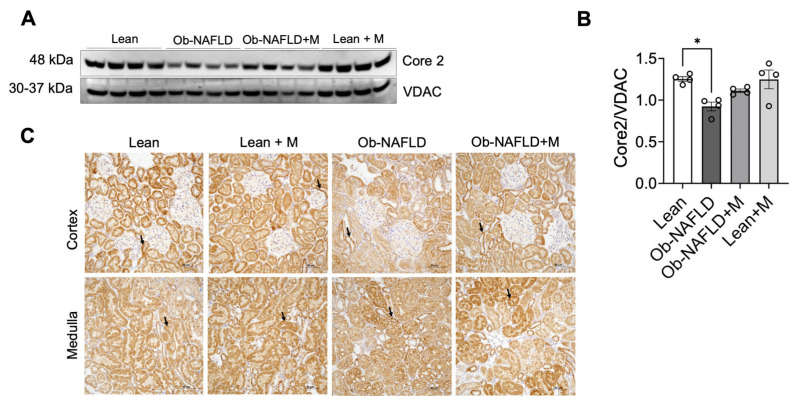
Renal mitochondrial Complex III (UQCRC2 subunit or Core 2) protein expression in obesity-associated NAFLD and the effect of metformin treatment. (**A**) Representative Western blot images showing renal expression of Core 2 in Lean, Ob-NAFLD, Ob-NAFLD+M, and Lean+M groups (*n* = 4 per group). VDAC was used as the mitochondrial loading control. (**B**) Densitometric quantification of Core 2 protein expression normalized to VDAC. Data are presented as mean ± SEM. Each dot represents an individual animal. Statistical significance was determined by two-way ANOVA (* *p* < 0.05). (**C**) Representative immunohistochemical staining of renal cortex and medulla sections showing Core 2 expression across experimental groups. Brown staining indicates positive immunoreactivity (arrows). Scale bars = 50 µm.

In contrast, marked alterations were observed at Complex III (Figure 3), a redox-sensitive site particularly susceptible to oxidative injury [43,44]. SDS-PAGE Western blotting revealed a significant reduction in the Core 2 subunit in obese kidneys compared with the lean controls (Figure 3A,B), supported by diminished tubular immunostaining (Figure 3C). Metformin modestly improved Complex III expression in obese rats; however, levels remained below those of lean controls in SDS-PAGE analysis, indicating incomplete restoration.

**Figure 4:**
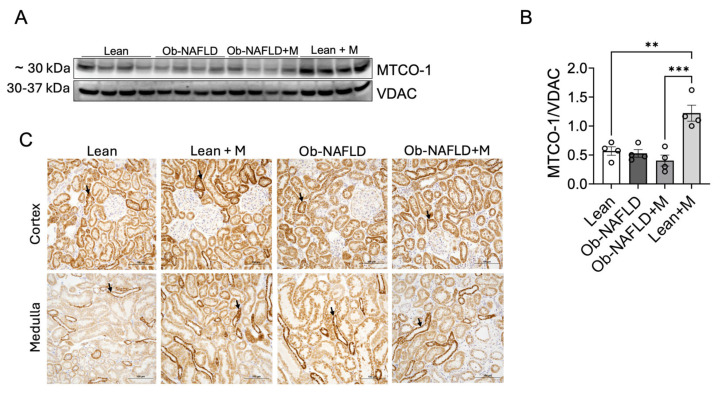
Renal mitochondrial Complex IV (MTCO-1 subunit) protein expression in Ob-NAFLD rats with or without metformin treatment. (**A**) Representative Western blot images showing renal expression of MTCO-1 in Lean, Ob-NAFLD, Ob-NAFLD+M, and Lean+M groups (*n* = 4 per group). VDAC was used as the mitochondrial loading control. (**B**) Densitometric quantification of MTCO1 protein expression normalized to VDAC. Data are presented as mean ± SEM. Each dot represents an individual animal. Statistical significance was determined by two-way ANOVA (** *p* < 0.01, *** *p* < 0.001). (**C**) Representative immunohistochemical staining of renal cortex and medulla sections showing MTCO1 expression across experimental groups. Brown staining indicates positive immunoreactivity (arrows). Scale bars = 100 µm.

Complex IV (MTCO1), which catalyzes electron transfer to molecular oxygen [15], remained relatively preserved (Figure 4). Both protein abundance (Figure 4A,B) and tubular distribution of MTCO1 (Figure 4C) were similar between lean and obese groups. Metformin increased renal MTCO1 expression in lean animals, suggesting enhanced mitochondrial oxidative capacity under normal conditions, whereas this response was absent in obese rats, indicating impaired adaptability under metabolic stress.

**Figure 5:**
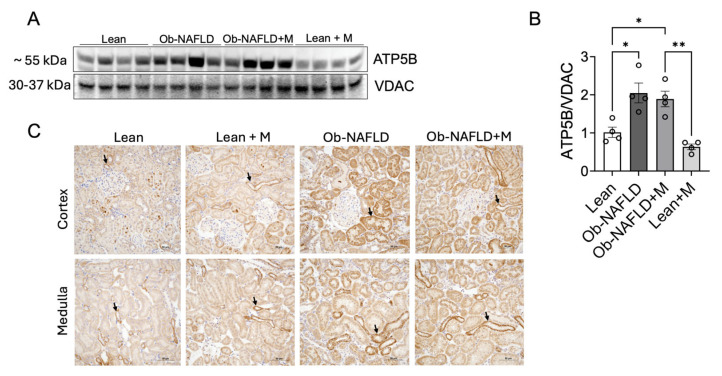
Renal mitochondrial Complex V (ATP5B) protein expression in Ob-NAFLD rats with or without metformin treatment. (**A**) Representative Western blot images showing renal expression of ATP5B in Lean, Ob-NAFLD, Ob-NAFLD+M, and Lean+M groups (*n* = 4 per group). VDAC was used as the mitochondrial loading control. (**B**) Densitometric quantification of ATP5B protein expression normalized to VDAC. Data are presented as mean ± SEM. Each dot represents an individual animal. Statistical significance was determined by two-way ANOVA (* *p* < 0.05, ** *p* < 0.01). (**C**) Representative immunohistochemical staining of renal cortex and medulla sections showing ATP5B expression across experimental groups. Brown staining indicates positive immunoreactivity (arrows). Scale bars = 50 µm.

At the level of ATP synthesis, ATP5B (Complex V subunit) protein levels were significantly elevated in obese kidneys compared with lean controls (Figure 5A,B), supported by increased tubular staining (Figure 5C). Metformin treatment did not fully normalize ATP5B expression in obese kidneys (Figure 5A–C).

### 3.2. BN-PAGE Analyses Reveal Dysregulated Assembly of Mitochondrial Complexes III and V

**Figure 6:**
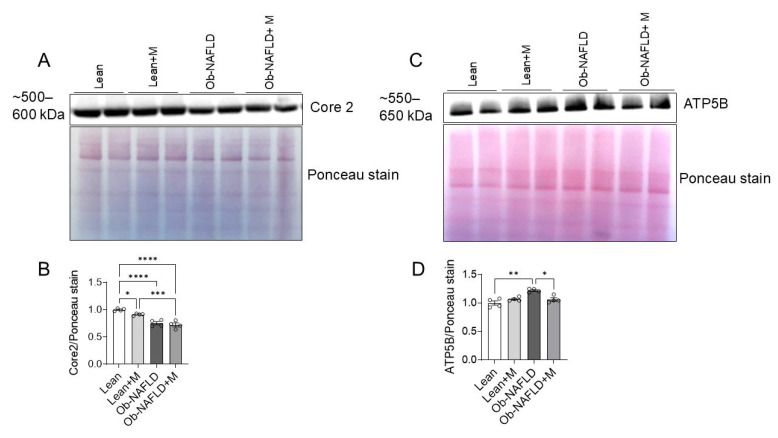
BN-PAGE Western blot analysis of mitochondrial Complex III and Complex V in renal tissue. (**A**) Representative BN-PAGE immunoblot showing expression of Core 2 (Complex III) across experimental groups: Lean, Lean+M, Ob-NAFLD, and Ob-NAFLD+M (*n* = 4 per group). Ponceau S staining was used as a loading control for normalization. (**B**) Densitometric quantification of Core 2 normalized to Ponceau S staining, indicating relative changes in Complex III abundance across groups. (**C**) Representative BN-PAGE immunoblot showing protein levels of ATP synthase subunit β (ATP5B; Complex V) in the same experimental groups. Ponceau S staining was used as a loading control for normalization. (**D**) Densitometric analysis of ATP5B normalized to Ponceau S staining showing relative abundance of Complex V across groups. Data are presented as mean ± SEM. Each dot represents an individual animal sample. Statistical significance was determined by two-way ANOVA (* *p* < 0.05, ** *p* < 0.01, *** *p* < 0.001, **** *p* < 0.0001).

Consistent with SDS-PAGE findings (Figure 1, Figure 2 and Figure 4), BN-PAGE analysis showed no significant changes in complex I (NDUFS3), complex II (SDHA), or complex IV (MTCO1), indicating preserved structure integrity. Importantly, native Complex III abundance was reduced in Ob-NAFLD kidneys (Figure 6A,B), suggesting impaired assembly or stability. BN-PAGE also confirmed increased native Complex V levels in Ob-NAFLD rats (Figure 6C,D), consistent with mitochondrial remodeling at the level of ATP synthase. Notably, metformin significantly reduced Complex V abundance in obese rats (Figure 6C,D), indicating partial correction of bioenergetic remodeling.

### 3.3. In-Gel Activity Assays Reveal Functional Remodeling of Complex V

**Figure 7:**
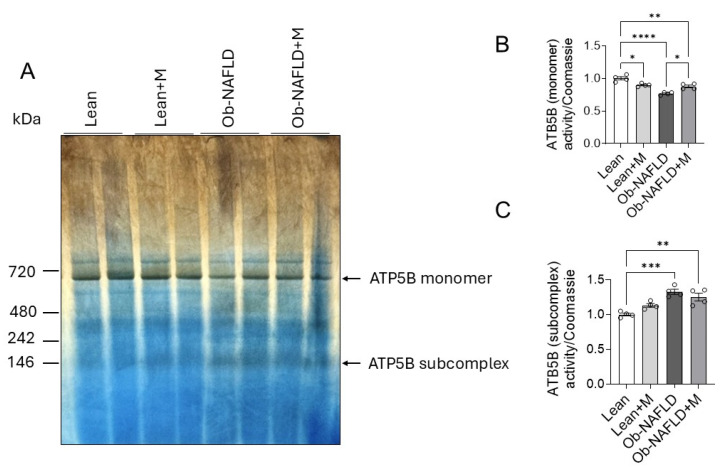
In-gel ATP synthase (Complex V) activity analysis. (**A**) Representative BN-PAGE (lauryl maltoside) in-gel ATP synthase activity assay showing Complex V activity across experimental groups: Lean, Lean+M, Ob-NAFLD, and Ob-NAFLD+M (*n* = 4 per group). The ~550–650 kDa band represents the ATP synthase holoenzyme, while the ~140 kDa band represents an ATP synthase assembly intermediate/subcomplex. Band intensity reflects enzymatic ATP hydrolysis activity of Complex V within native mitochondrial complexes. (**B**) Quantification of ATP5B monomer activity normalized to Coomassie staining, demonstrating relative changes in monomeric Complex V activity among groups. (**C**) Quantification of ATP5B subcomplex activity normalized to Coomassie staining, showing alterations in higher-order Complex V assembly-associated activity across experimental conditions. Data are presented as mean ± SEM. Each dot represents an individual animal sample. Statistical significance was determined by two-way ANOVA (* *p* < 0.05, ** *p* < 0.01, *** *p* < 0.001, **** *p* < 0.0001).

In-gel activity assays demonstrated that the monomeric form of Complex V, the primary contributor of ATP production, exhibited significantly reduced activity in Ob-NAFLD rats compared with lean controls (Figure 7A,B), indicating impaired functional efficiency. In this context, the “monomer” refers to the fully assembled ATP synthase holoenzyme (~550–650 kDa) detected under native BN-PAGE conditions. Metformin significantly restored monomer-associated Complex V activity in Ob-NAFLD kidneys, suggesting partial functional recovery.

In contrast, the Complex V subcomplex (assembly intermediate) exhibited increased in-gel activity in Ob-NAFLD rats (Figure 7A,C), indicating enhanced accumulation of partially assembled ATP synthase. This subcomplex corresponds to a lower-molecular-weight ATP synthase assembly intermediate (~140 kDa) that lacks the complete structural organization of the mature holoenzyme. This abnormality persisted after metformin treatment, indicating incomplete reversal of assembly defects.

Collectively, these findings demonstrate a shift toward dysfunctional ATP synthase assembly, characterized by reduced holoenzyme activity and increased subcomplex accumulation, with metformin selectively improving functional activity but not assembly integrity.

### 3.4. Obesity-Associated NAFLD Drives Mitochondrial Fragmentation and Fusion Impairment

**Figure 8:**
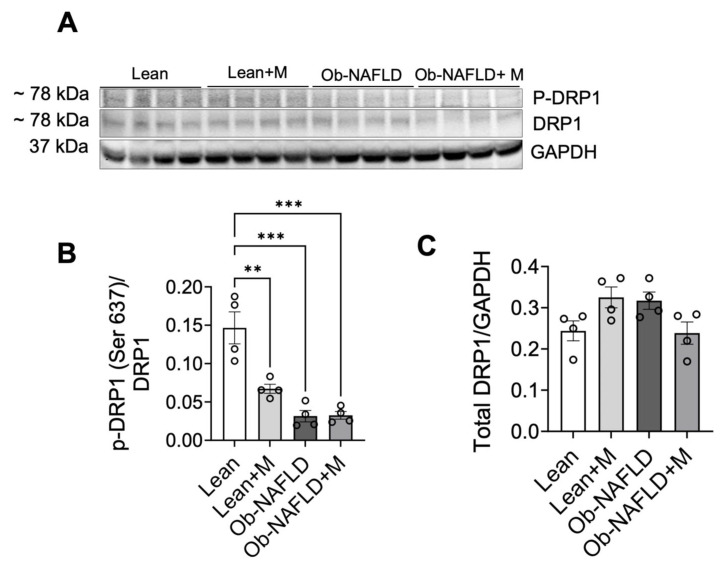
Renal DRP1 phosphorylation (inhibitory) and total DRP1 expression in obesity-associated NAFLD and the effect of metformin treatment. (**A**) Representative Western blots showing phosphorylated DRP1 (p-DRP1, Ser637), total DRP1, and GAPDH across the four experimental groups: Lean, Lean+M, Ob-NAFLD, and Ob-NAFLD+M (*n* = 4 per group). (**B**) Quantification of p-DRP1 (Ser637) normalized to total DRP1. (**C**) Quantification of total DRP1 normalized to GAPDH. Total DRP1 expression remains relatively unchanged across groups. Data are presented as mean ± SEM. Each dot represents an individual animal. Statistical significance was determined by two-way ANOVA (** *p* < 0.01, *** *p* < 0.001).

Because mitochondrial function is closely linked to morphology, we examined regulators of mitochondrial dynamics. DRP1, a key mediator of mitochondrial fission, is activated by reduced phosphorylation at Ser637 [45]. Obese rats exhibited significantly reduced DRP1 Ser637 phosphorylation compared with lean controls (Figure 8A,B), indicating enhanced fission. Total DRP1 levels were unchanged (Figure 8A–C), suggesting post-translational regulation. Metformin significantly reduced phospho-DRP1 levels in lean rats but had no effect on obese animals (Figure 8A,B), suggesting impaired responsiveness under metabolic stress.

**Figure 9:**
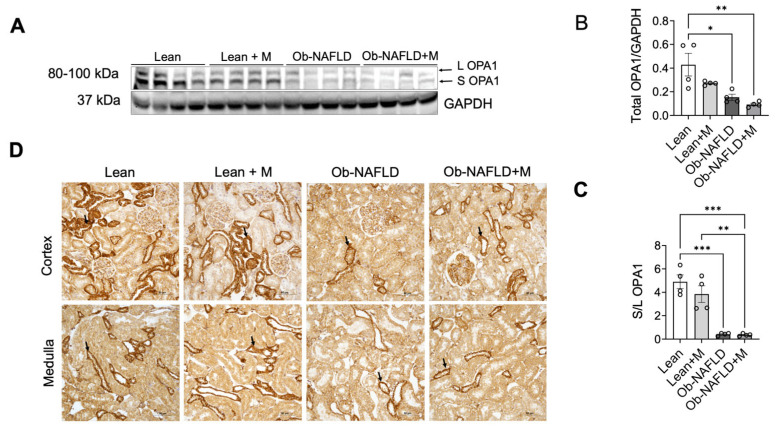
Alterations in OPA1 expression and processing in kidney tissues. (**A**) Representative immunoblot showing long (L-OPA1) and short (S-OPA1) isoforms of OPA1 in the samples from Lean, Lean+M, Ob-NAFLD, and Ob-NAFLD+M groups (*n* = 4 per group). GAPDH was used as a loading control. (**B**) Quantification of total OPA1 normalized to GAPDH. (**C**) Ratio of short (S) to long (L) OPA1 isoforms (S/L OPA1). (**D**) Representative immunohistochemical staining for OPA1 in kidney cortex and medulla from the study groups. Brown staining indicates OPA1 immunoreactivity (arrows), demonstrating the localization of OPA1 within renal tubular epithelial cells. Data are presented as mean ± SEM with individual data points shown. Statistical significance was determined by two-way ANOVA (* *p* < 0.05, ** *p* < 0.01, *** *p* < 0.001). Scale bars = 50 µm.

OPA1, essential for inner membrane fusion and cristae integrity [28,29,46], was significantly reduced in obese kidneys (Figure 9A,B). The OPA1 short-to-long (S/L) ratio was also significantly decreased in obese kidneys (Figure 9C), indicating impaired fusion capacity. Metformin did not affect total OPA1 levels or the S/L processing. Immunohistochemical analysis confirmed preserved tubular localization of OPA1 across all groups (Figure 9D). Together, these findings indicate a shift toward increased fission and impaired fusion in Ob-NAFLD kidneys.

### 3.5. Ob-NAFLD Promotes ATP Overproduction with Partial Normalization by Metformin

**Figure 10:**
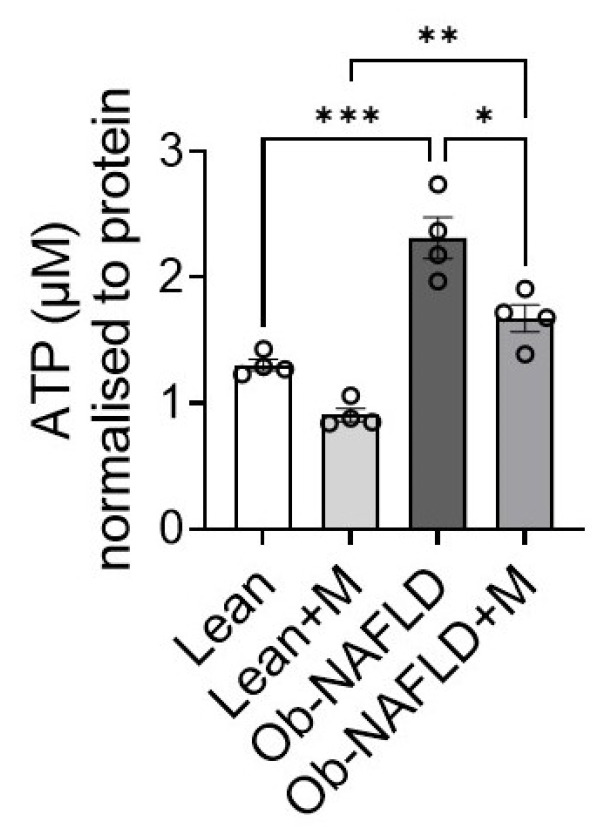
ATP levels in the kidney tissues from Lean, Lean+M, Ob-NAFLD, and Ob-NAFLD+M groups (*n* = 4 per group). ATP concentrations (µM) normalized to protein in kidney samples. Data are presented as mean ± SEM with individual data points shown. Statistical significance was determined by two-way ANOVA (* *p* < 0.05, ** *p* < 0.01, *** *p* < 0.001).

Renal ATP levels were significantly elevated in Ob-NAFLD kidneys compared with lean controls (Figure 10). Metformin partially normalized ATP levels in Ob-NAFLD rats, although values remained above baseline. ATP levels in lean animals were unchanged, reflecting tight bioenergetic regulation under normal conditions.

### 3.6. Renal ATPase Inhibitory Factor 1 (IF1) Remains Unchanged Despite Mitochondrial Stress

**Figure 11:**
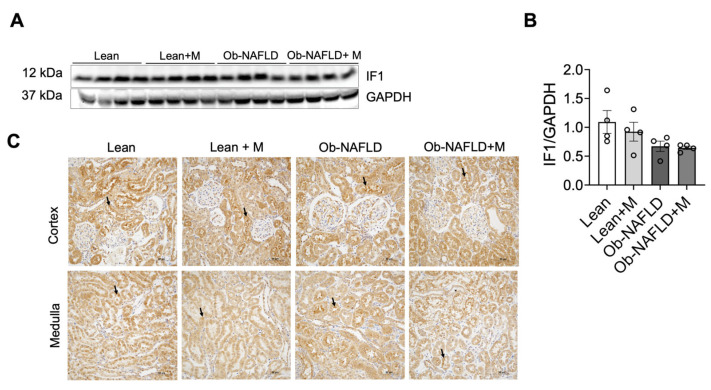
Renal ATPase IF1 expression in Ob-NAFLD rats with or without metformin treatment. (**A**) Representative immunoblot analysis showing IF1 protein levels in kidney samples from Lean, Lean+M, Ob-NAFLD, and Ob-NAFLD+M groups (*n* = 4 per group). GAPDH was used as a loading control. (**B**) Quantification of IF1 protein normalized to GAPDH. Data are presented as mean ± SEM with individual data points shown. Each dot represents an individual animal. (**C**) Representative immunohistochemical staining of IF1 in kidney cortex and medulla from the groups. Brown staining indicates IF1 localization (arrows). Scale bars = 50 µm.

IF1, a regulator that limits ATP hydrolysis under stress, was assessed to determine its role in mitochondrial adaptation. Surprisingly, renal IF1 protein levels remained unchanged across all groups (Figure 11A,B), with consistent tubular localization (Figure 11C). This stability suggests that increased ATP production in Ob-NAFLD kidneys occurs independently of IF1 regulation. The absence of IF1 modulation may contribute to sustained energetic imbalance and persistent tubular stress, providing insight into the incomplete reno-protective effects of metformin [5]. 

Full-length, uncropped immunoblot images corresponding to all Western blot figures are provided as Supplementary Figures S1–S11 (Supplementary Materials).

## 4. Discussion

Ob-NAFLD imposes a chronic metabolic burden that extends beyond the liver and disrupts renal mitochondrial homeostasis [5,23,24]. Using a well-established rat model of Ob-NAFLD, we demonstrated that renal mitochondrial dysfunction in this setting is not characterized by global energetic failure, but rather by selective ETC remodeling, dysregulated mitochondrial dynamics, and compensatory yet inefficient ATP overproduction. Although metformin partially alleviates this bioenergetic stress, it does not fully restore mitochondrial structural integrity or regulatory balance.

Renal tubular cells rely heavily on oxidative phosphorylation to maintain solute transport and cellular homeostasis, rendering the kidney particularly vulnerable to metabolic stress/overload [7]. Consistent with previous reports [23,30,47], we observed selective rather than global ETC alterations, indicating preserved overall respiratory capacity alongside targeted vulnerability of specific complexes. This pattern supports the emerging concept that metabolic disease preferentially disrupts redox-sensitive ETC nodes, rather than causing global mitochondrial failure [48,49].

Specifically, Complexes I, II, and IV remained largely intact, whereas complex III was significantly reduced, identifying a focal defect at a critical redox hub of the ETC. Given its central role in electron transfer from ubiquinol to cytochrome c, impairment of Complex III likely promotes electron leakage, increased ROS generation, and reduced bioenergetic efficiency [43]. These findings are consistent with studies showing that Complex III dysfunction is a major contributor to oxidative stress in obesity and insulin resistance [50,51]. Importantly, this defect may destabilize overall respiratory chain function even when upstream complexes remain preserved [52], indicating that mitochondrial dysfunction in obesity reflects functional reorganization rather than loss of mitochondrial content.

In contrast, Complex V exhibited increased expression and activity, accompanied by elevated renal ATP levels. While increased ATP content is often interpreted as preserved mitochondrial function, accumulating evidence suggests that this reflects compensatory ATP synthase hyperactivation in response to upstream ETC inefficiencies [53,54]. When electron flow is constrained at Complex III, increased ATP synthase activity may help maintain energy output but at the cost of reduced coupling efficiency and increased energetic stress [24,55]. Our findings further indicate that this response involves altered ATP synthase assembly, favoring accumulation of subcomplex forms over fully functional monomers, thereby contributing to inefficient ATP production.

Metformin partially corrected this imbalance by modestly restoring Complex III expression and reducing excessive Complex V upregulation. These results align with previous reports showing that metformin improves mitochondrial efficiency through AMPK activation and reduces ROS production rather than complete restoration of ETC architecture [56,57,58]. However, the incomplete normalization observed here suggests that chronic obesity induces semi-stable mitochondrial remodeling that is only partially reversible with metabolic therapy.

A notable observation is the stability of IF1 despite significant alterations in ATP levels and ETC organization. IF1 normally acts as a protective regulator by limiting ATP synthase reversal during stress [59]. Its unchanged expression suggests either a ceiling effect or a failure to engage this regulatory checkpoint under chronic metabolic stress. This lack of IF1 modulation may permit sustained mitochondrial overactivity, contributing to persistent energetic imbalance [60,61]. Importantly, metformin did not influence IF1 levels, indicating that its reno-protective effects occur independently of this pathway [5].

Structural remodeling of mitochondria further reinforces these functional disturbances. Increased DRP1 activation and reduced OPA1 expression indicate a shift toward mitochondrial fission and impaired fusion. Reduced inhibitory phosphorylation of DRP1, combined with decreased OPA1 abundance and altered isoform balance, likely drives mitochondrial fragmentation, cristae disruption, and increased susceptibility to oxidative stress and apoptosis. This changes are consistent with prior studies linking mitochondrial dynamics dysregulation to renal injury in obesity and NAFLD [62,63,64,65]. Importantly, metformin failed to reverse these alterations, suggesting that mitochondrial structural remodeling becomes established during chronic metabolic stress.

This study has several limitations that should be considered when interpreting the findings. Only female Zucker rats were used, which limits our understanding of potential sex-related differences in metabolic kidney injury. In addition, the relatively young age of the animals makes it difficult to fully relate the findings to aging-associated chronic kidney disease. The model also reflects a combined obesity–NAFLD condition, so it is not possible to clearly distinguish the individual contributions of obesity and NAFLD to kidney injury. Mechanistic validation is limited, as no in vitro experiments or interventional approaches (such as DRP1 inhibition) were included to confirm causality. Multi-omics analyses were also not performed, and key stress pathways, including ER stress and cellular senescence, were not assessed. Finally, the study is largely observational, which limits strong causal conclusions about mitochondrial dysfunction and renal injury. Despite these limitations, the study provides useful in vivo evidence of renal mitochondrial alterations in obesity-associated NAFLD.

Collectively, our findings support a model in which Ob-NAFLD induces a hierarchical mitochondrial defect in the kidney characterized by (i) selective ETC remodeling centered on Complex III, (ii) compensatory but inefficient activation and disassembly of ATP synthase (Complex V), and (iii) persistent disruption of mitochondrial fusion–fission balance. While metformin partially improves bioenergetic imbalance, it does not fully restore mitochondrial architecture or regulatory control.

## 5. Conclusions

This study demonstrates that metabolic disease reshapes renal mitochondrial organization in a selective and initially adaptive, but ultimately maladaptive, manner. These findings suggest that therapies targeting ETC stability, mitochondrial dynamics, and energy regulatory checkpoints may be required to fully restore mitochondrial homeostasis and prevent progression of Ob-NAFLD-associated CKD.

## Figures and Tables

**Table 1:** Primary and secondary antibodies.

Antibody	Source	Dilution	Catalog Number
OPA1	Abcam	1:1000 WB, 1:100 IHC	ab42364
DRP1	Abcam	1:1000	ab56788
p-DRP1 (S637)	Abcam	1:1000	ab193216
NDUFS3	Abcam	1:1000 WB, 1:50 IHC	ab110246
SDHA	Abcam	1:1000 WB, 1:750 IHC	ab14715
UQCRC2	Abcam	1:1000 WB, 1:200 IHC	ab14745
MTCO-1	Abcam	1:1000 WB, 1:2000 IHC	Ab14705
ATP5B	Invitrogen	1:1000 WB, 1:100 IHC	PA5-81952
ATPIF1	Cell Signaling	1:1000 WB, 1:200 IHC	8528
VDAC	Abcam	1:1000 WB	ab14734
GAPDH	Signalway	1:1000 WB	SAB 37985
Peroxidase Goat Anti-Mouse IgG	Jackson Immuno Research	1:30,000 WB	115-035-166
Peroxidase Goat Anti-Rabbit IgG	Jackson Immuno Research	1:30,000 WB	111-035-144

## Data Availability

The original contributions presented in this study are included in the article. Further inquiries can be directed to the corresponding author.

## Supplementary Materials

The following supporting information can be downloaded at: https://www.mdpi.com/article/10.3390/nu18132061/s1, Figures S1–S11: Raw images.

## Abbreviations

The following abbreviations are used in this manuscript:

NAFLD	Non-alcoholic fatty liver disease
CKD	Chronic kidney disease
Ob	Obesity
ETC	Electron Transport Chain
ATP	Adenosine Triphosphate
M	Metformin
MEB	Mitochondria Extraction Buffer
PVDF	Polyvinylidene Difluoride
OPA1	Optic Atrophy 1
VDAC	Voltage-dependent anion channels
