# Supplementary material for: Impaired Renal Mitochondria and Bioenergetics During Obesity-Associated NAFLD
Source: Nutrients. 2026 Jun 24;18(13):2061. doi: 10.3390/nu18132061 (PMC13362658; doi:10.3390/nu18132061)

Figure S1: In-gel ATP synthase activity assay

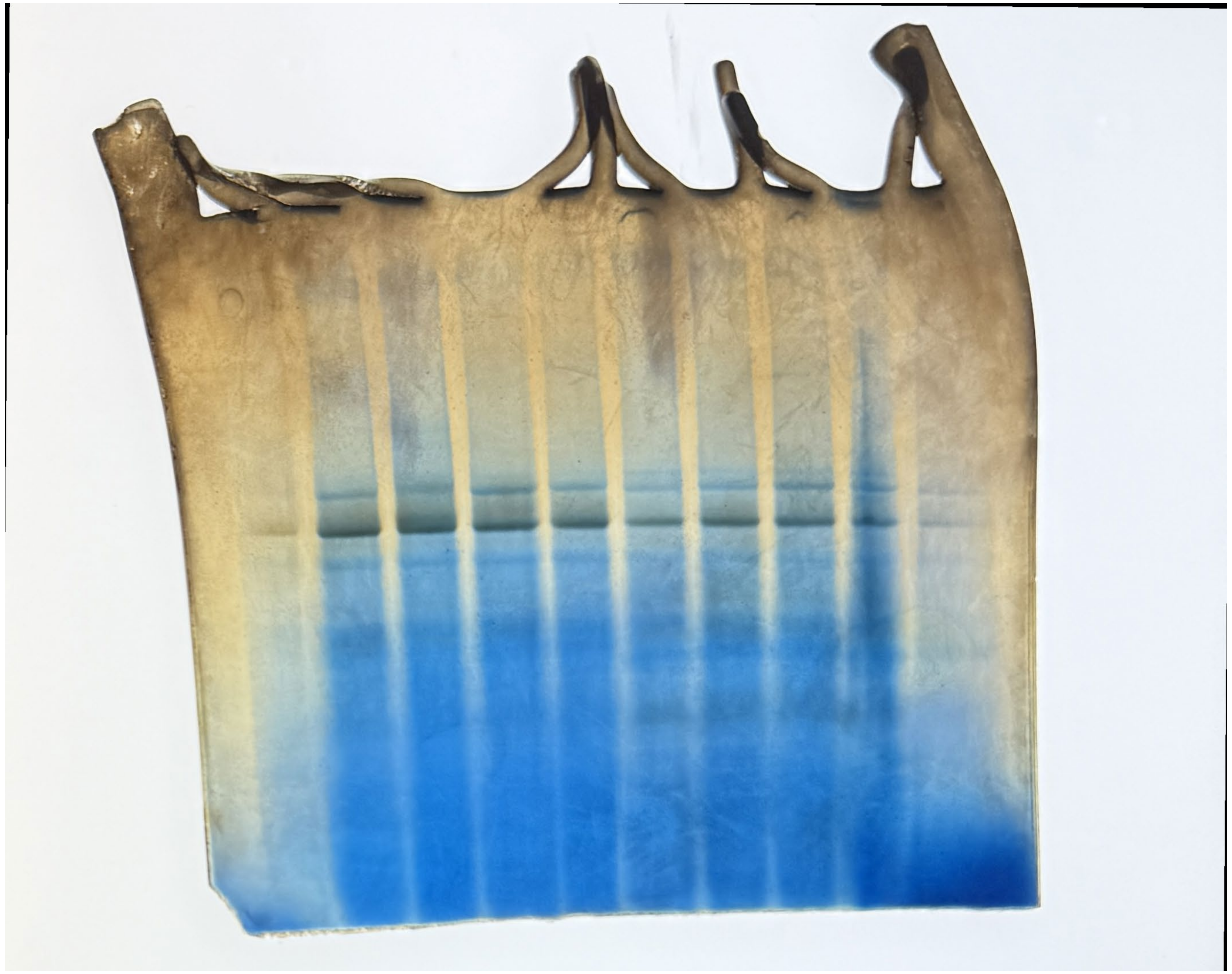

Gel 1

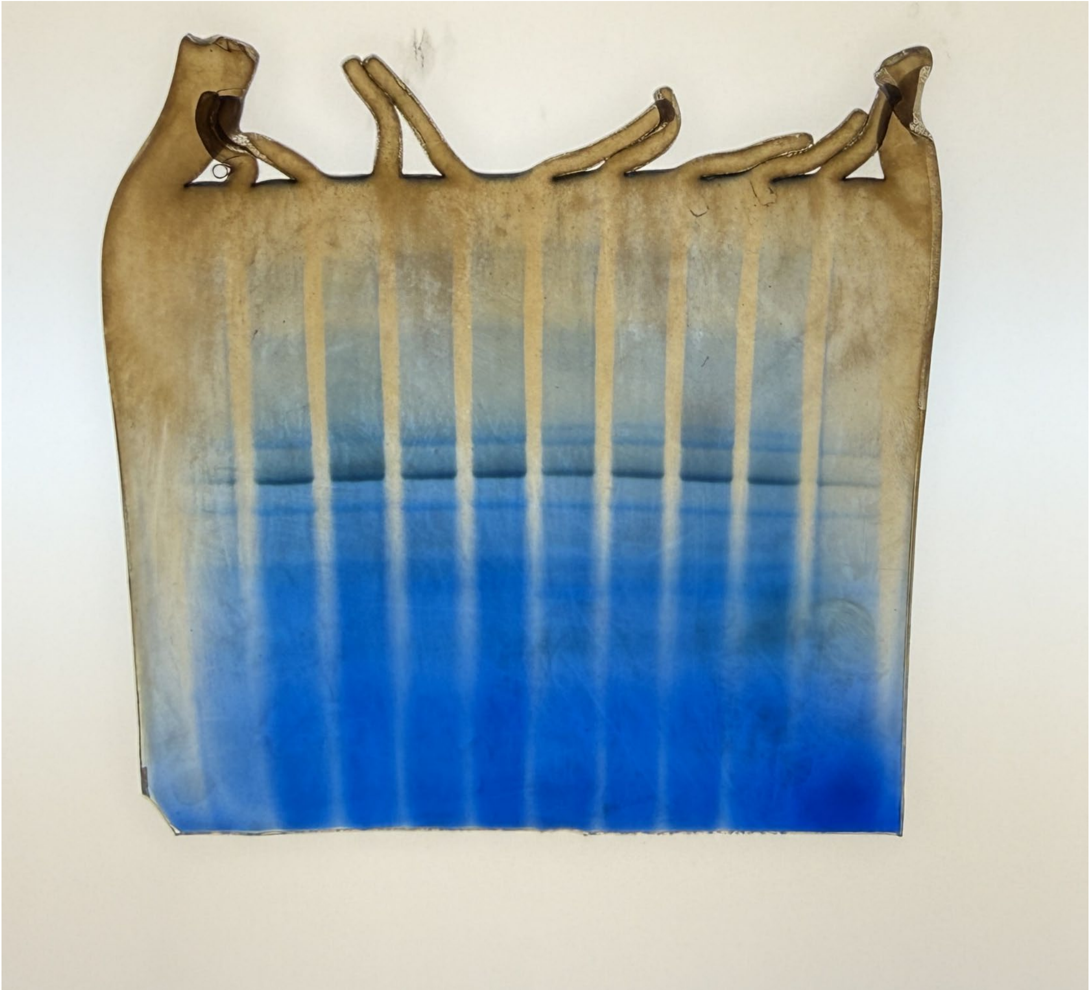

Gel 2

Figure S2: Western blot, BN-PAGE (lauryl maltoside)

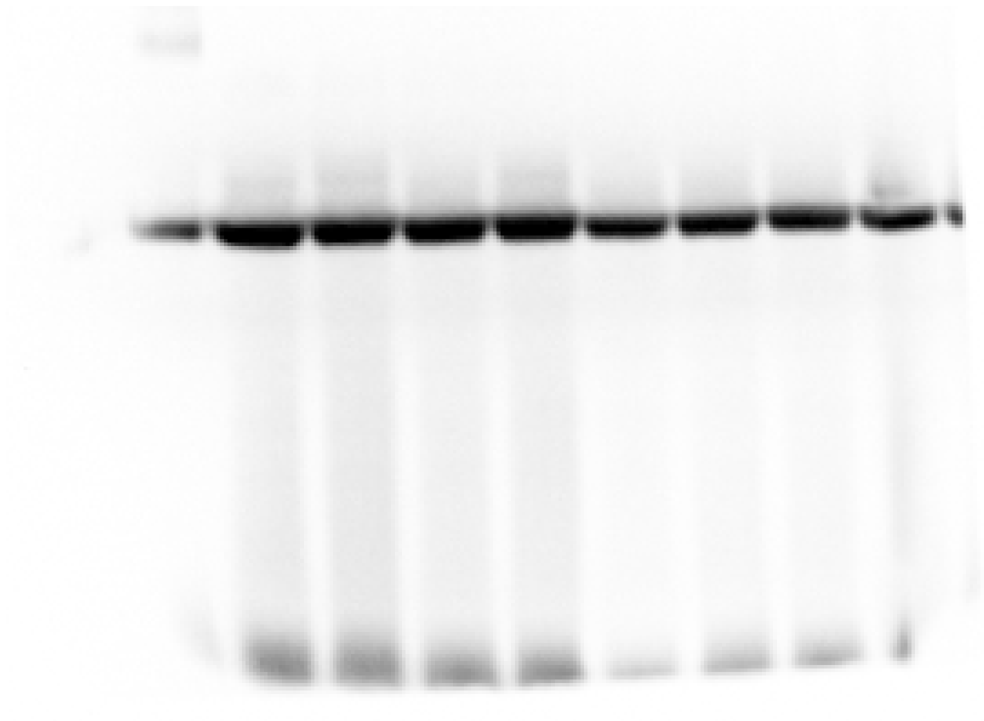

Gel 1

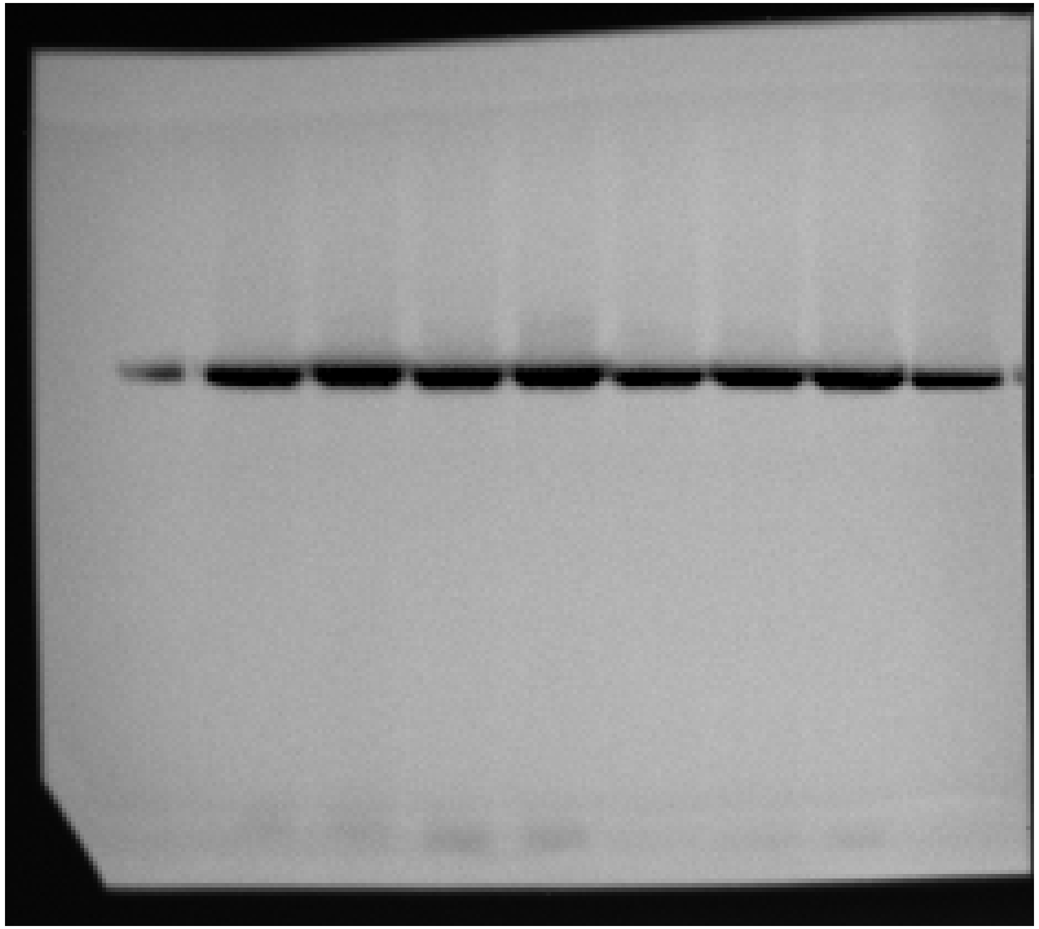

Gel 2

Figure S3: Western blot, BN-PAGE (lauryl maltoside)

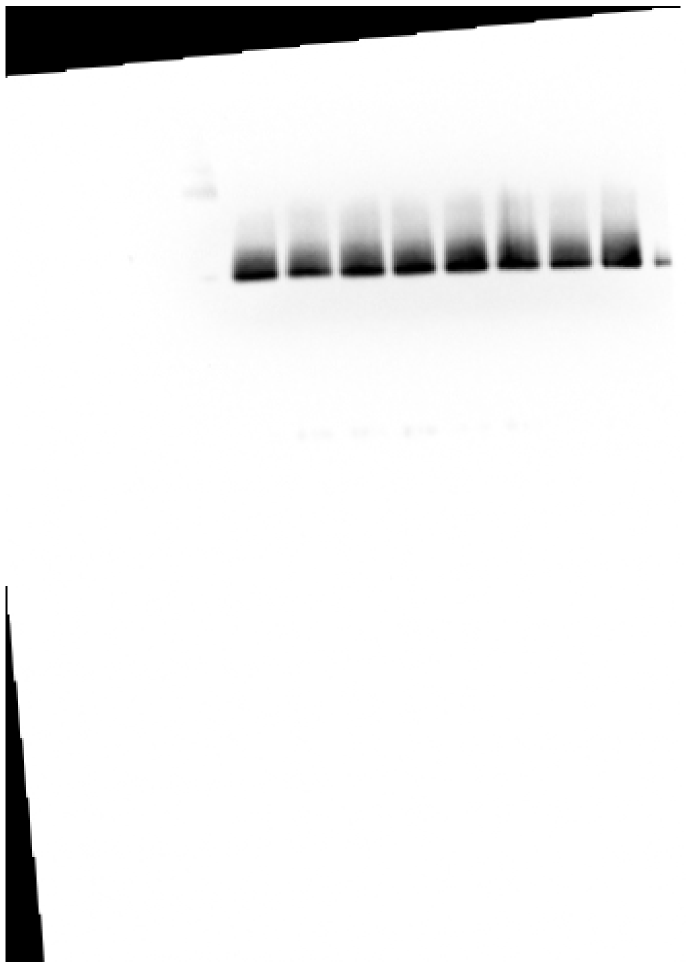

Gel 1

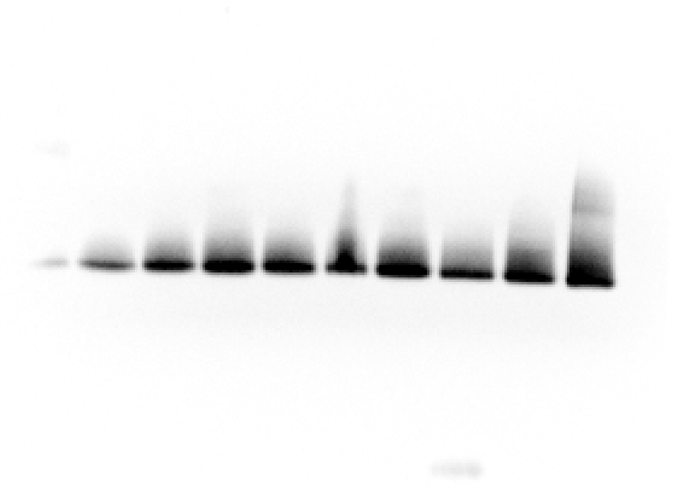

Gel 2

Figure S4: Western Blot, DRP1 and P-DRP1

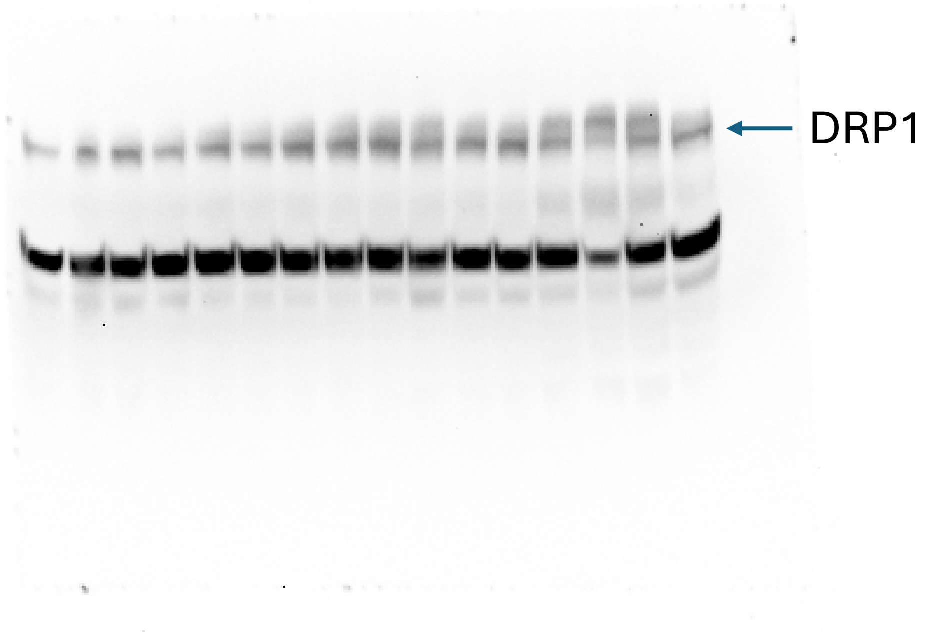

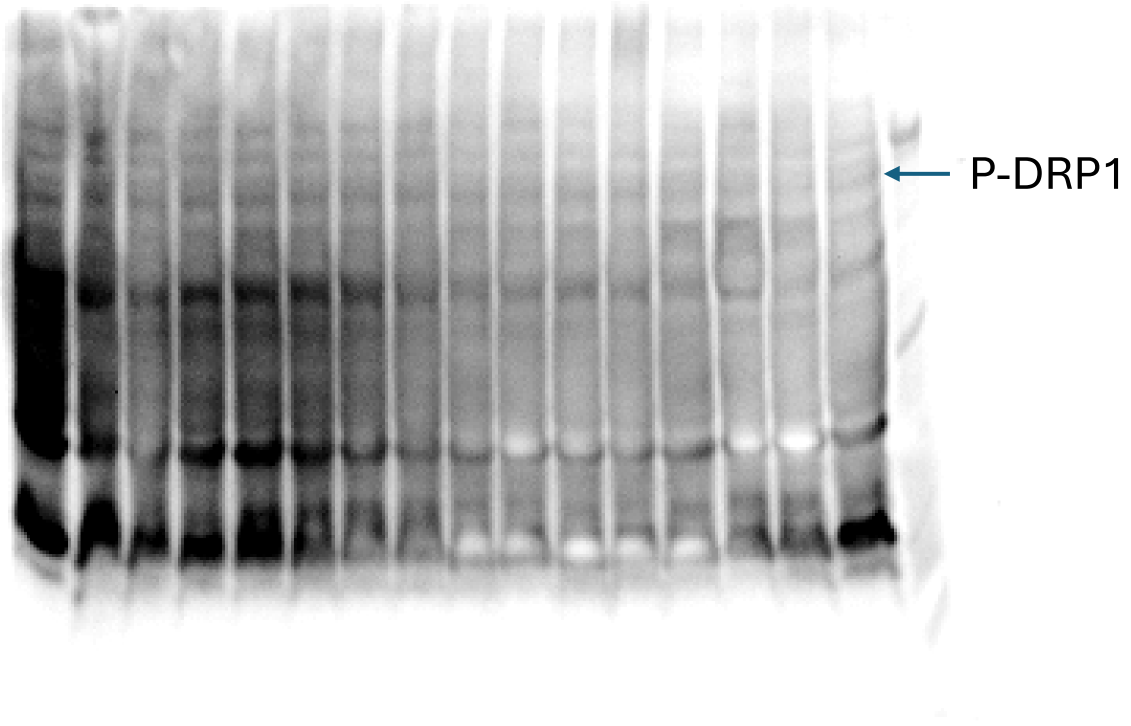

Figure S5: Western Blot, GAPDH

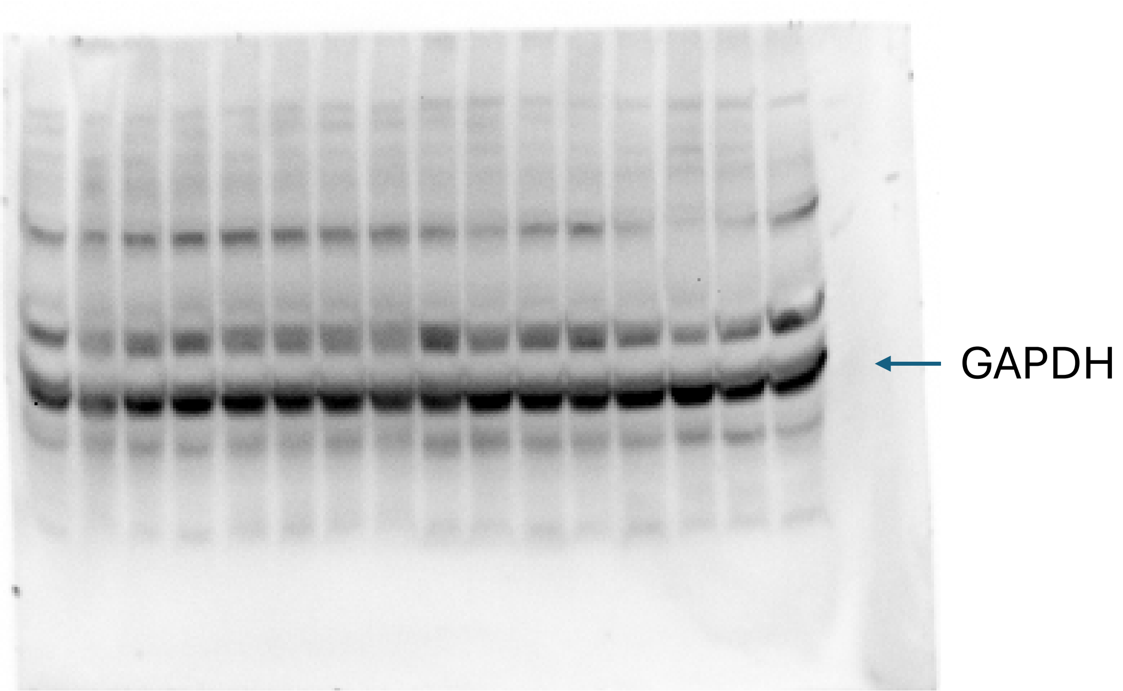

Figure S6: Western Blot, OPA1

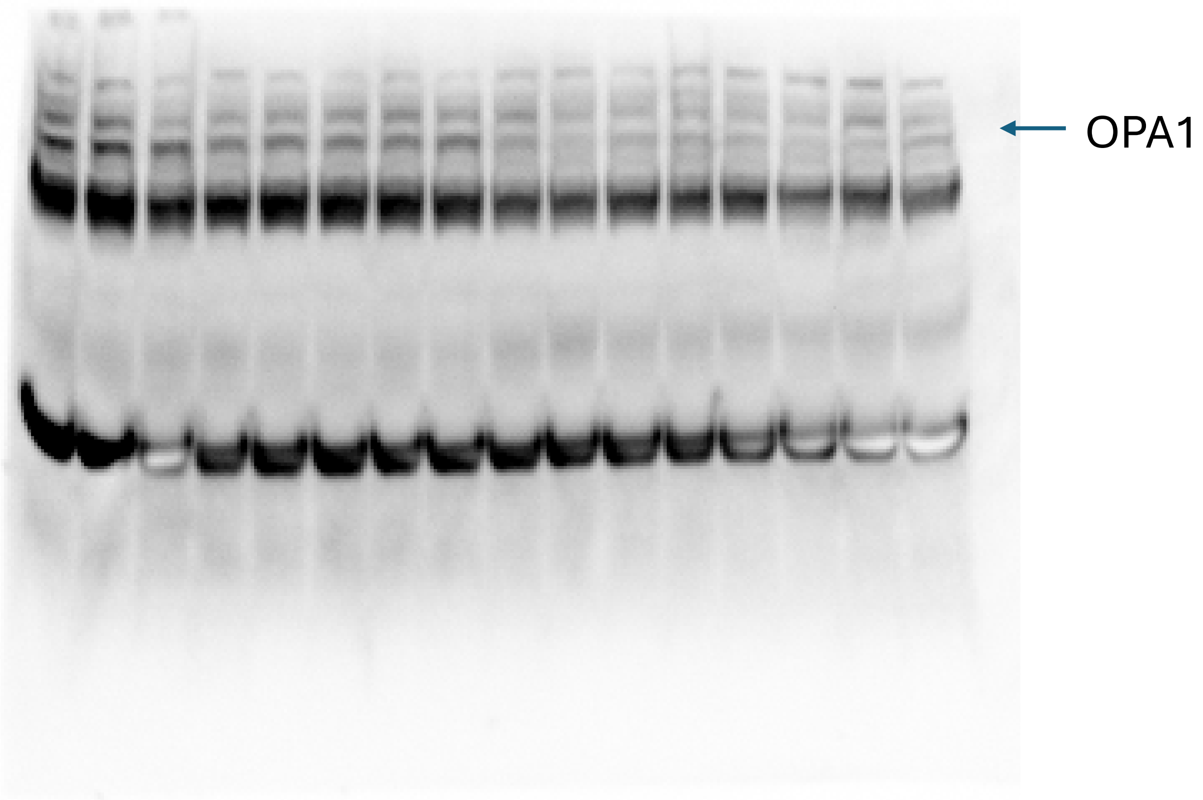

Figure S7: Western Blot, ATPIF1

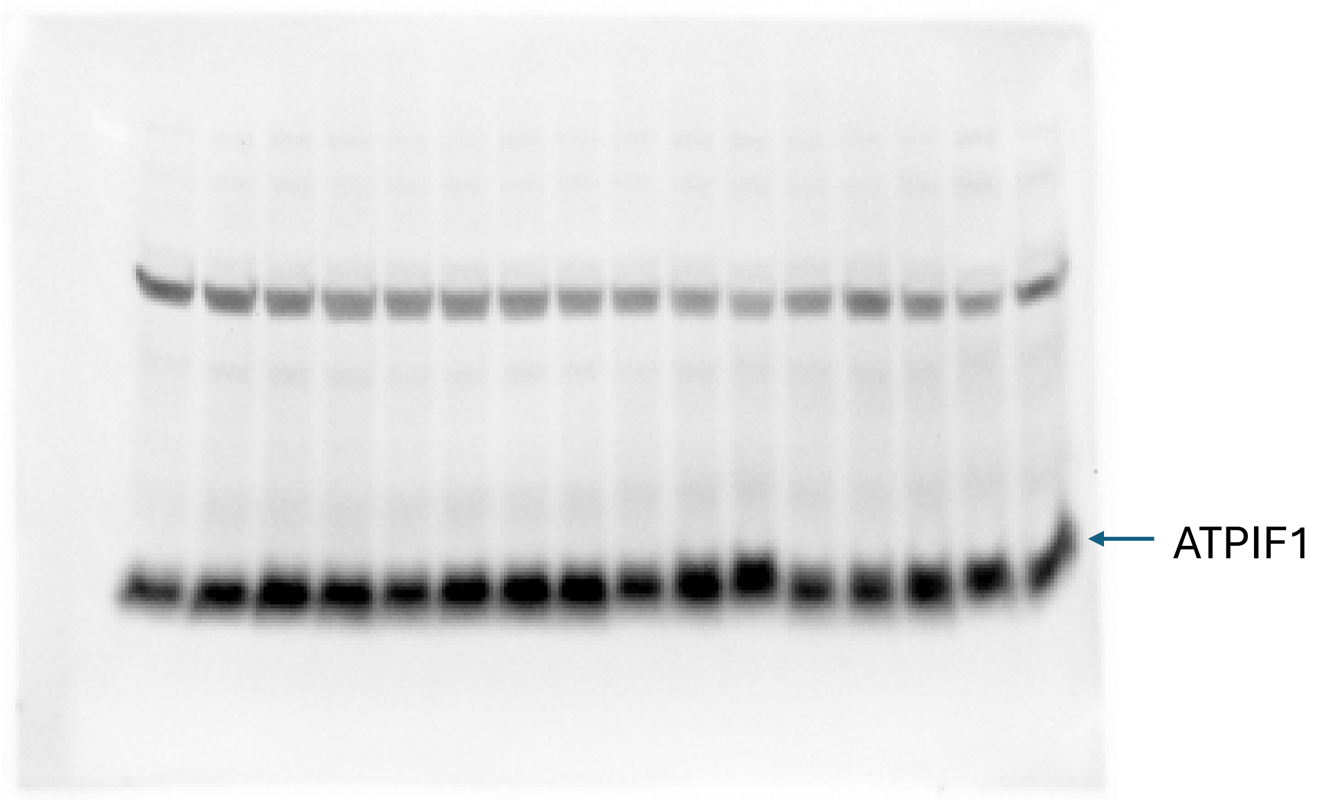

Figure S8: Western Blot, ATP5B

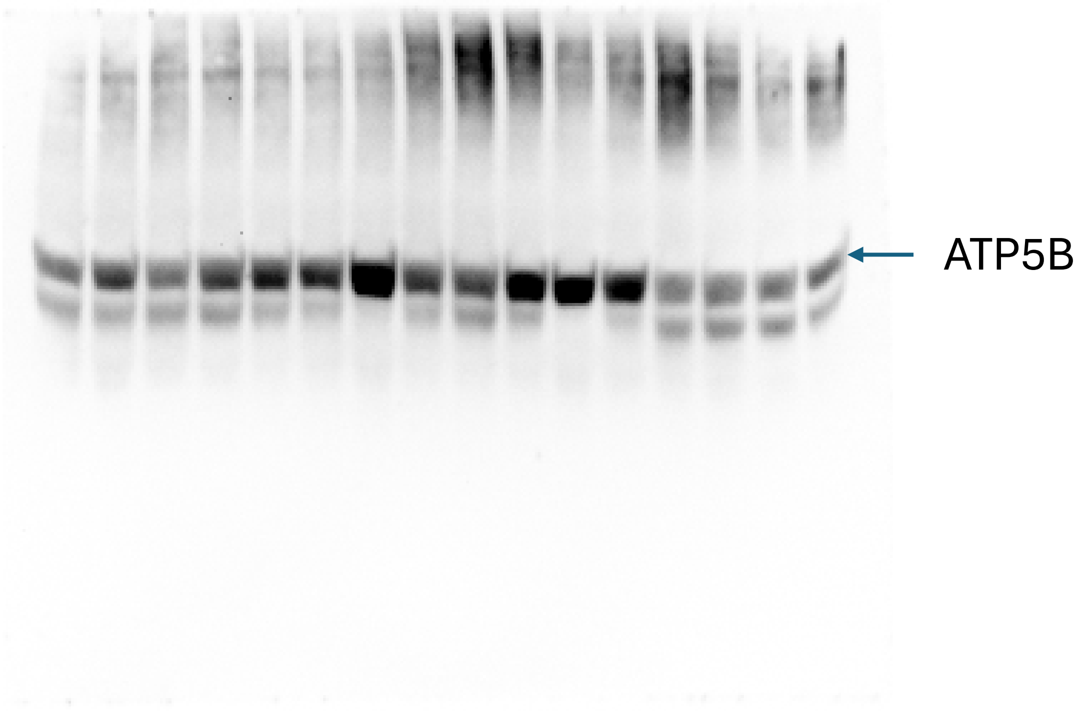

Figure S9: Western Blot, Core2

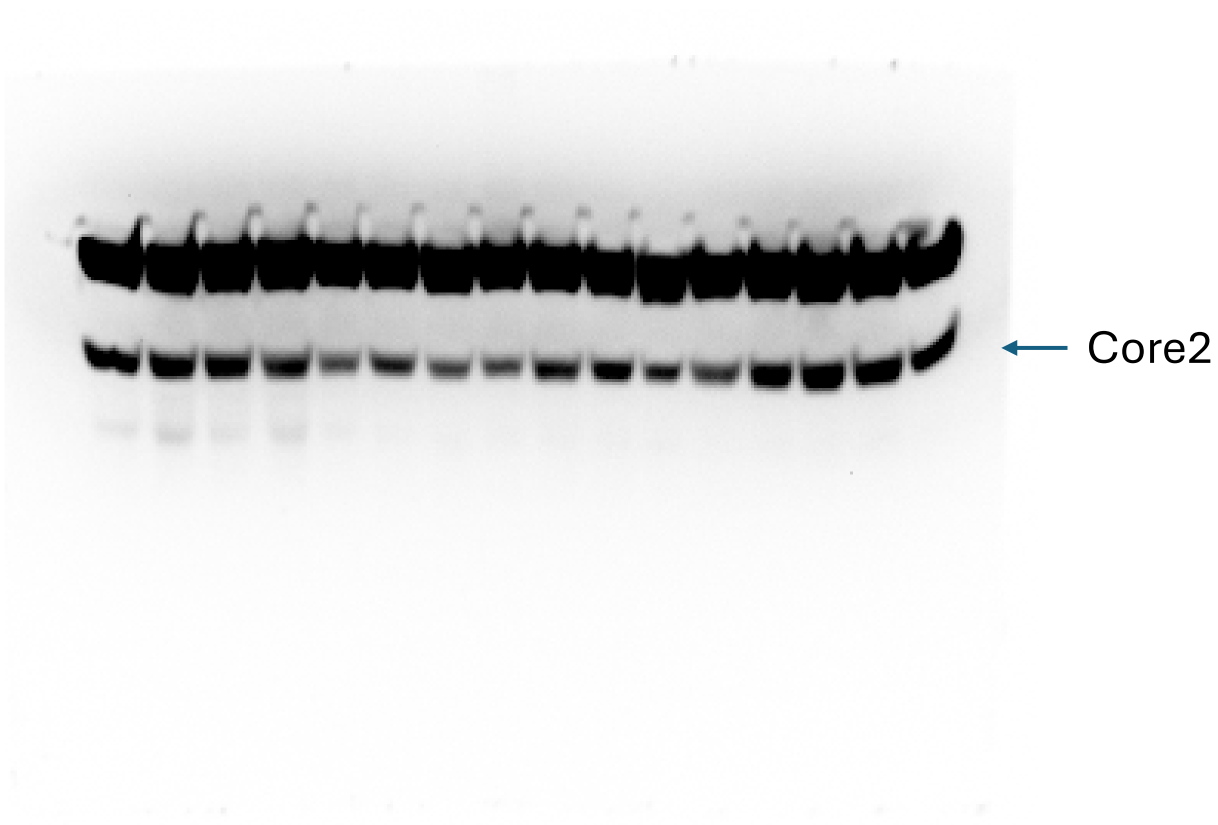

Figure S10: Western Blot, NDUFS3

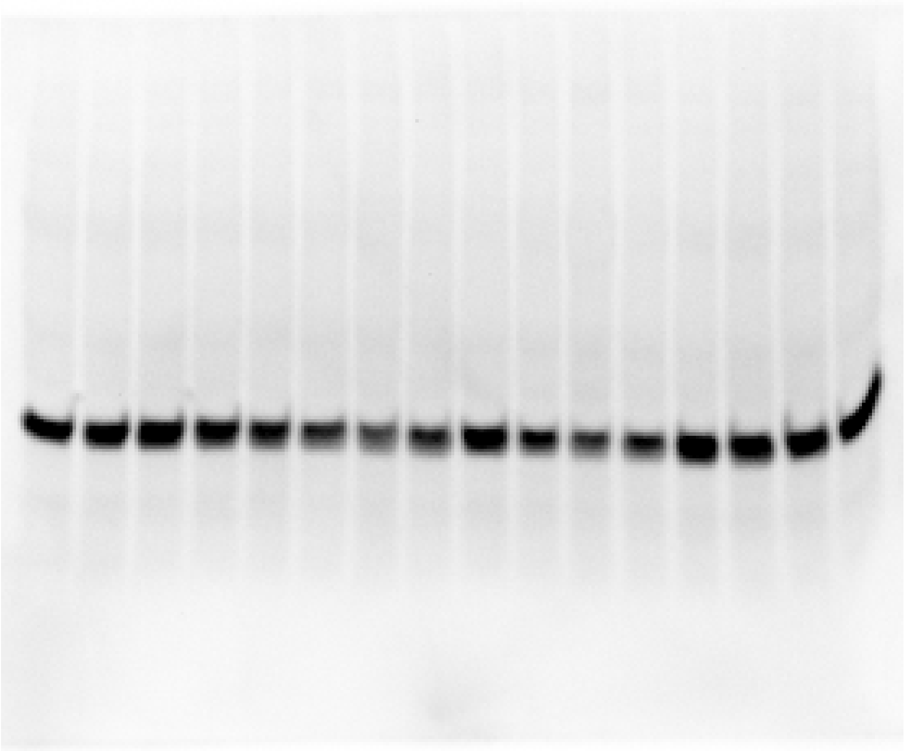

Figure S11: Western Blot, MTCO1

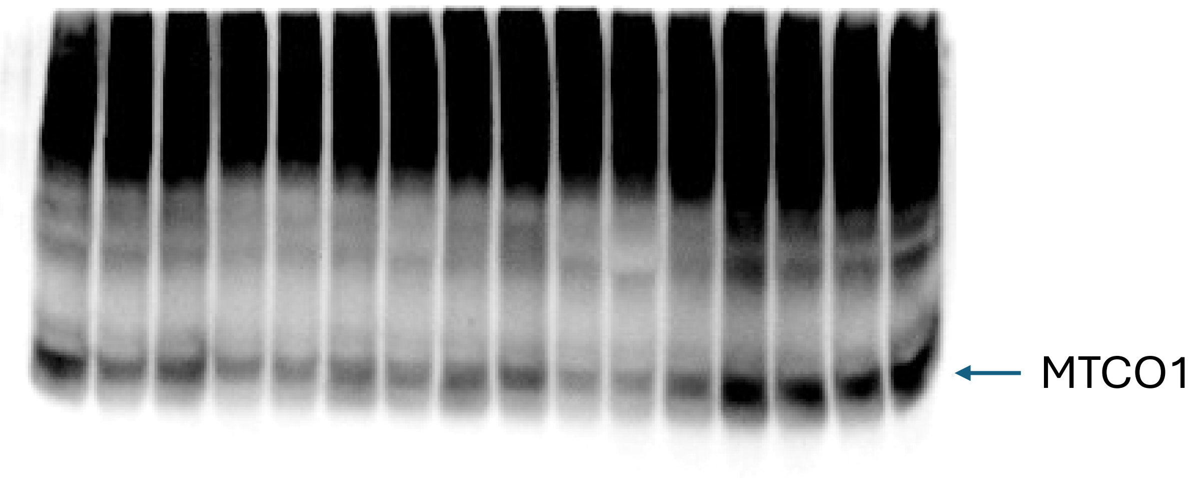

Supplement: Supplementary file 1 [file nutrients-18-02061-s001.zip › nutrients-4360734-supplementary.pdf]
